# Feasibility of MRI targeted single fraction HDR brachytherapy for localized prostate carcinoma: ProFocAL-study

**DOI:** 10.1007/s00432-022-04491-3

**Published:** 2022-11-29

**Authors:** Peter Hass, Frank Fischbach, Maciej Pech, Ahmed Gawish

**Affiliations:** 1 Department of Radiation Oncology, Otto-von-Guericke University, Leipziger Str. 44, 39120 Magdeburg, Germany; 2 Department of Radiology and Nuclear Medicine, Otto-von-Guericke University, Magdeburg, Germany; 3 Department of Radiation Oncology, Helios Hospital Erfurt, Erfurt, Germany

**Keywords:** Brachytherapy, Prostate cancer, Focal therapy, Biochemical failure, MRI

## Abstract

**Purpose:**

A potential method for focal therapy in locally advanced prostate cancer is focal brachytherapy (F-BT). The purpose of this research was to evaluate midterm F-BT oncologic, functional, and toxicological results in men who had therapy for prostate cancer.

**Materials and methods:**

Between 2016 and 2020, F-BT was used to treat 37 patients with low- to intermediate-risk prostate cancer. The recommended dosage was 20 Gy. Failure was defined as the existence of any prostate cancer that has persisted in-field after treatment. The F-BT oncologic and functional outcomes served as the main and secondary objectives, respectively.

**Results:**

A median 20-month follow-up (range 14–48 months). 37 patients received F-BT and enrolled in the study; no patient experienced a biochemical recurrence in the first 24 months, according to Phoenix criteria. In the control biopsies, only 6 patients showed in-field failure. The median initial IPSS was 6.5, at 6 months was 6.0, and at 24 months was 5.0. When the median ICIQ-SF score was 0 at the baseline, it remained 0 at 6-, 12-, and 24 months. Overall survival and biochemical disease-free survival after 3 years were all at 100% and 86.4%, respectively. There was no notable acute gastro-intestinal (GI) or genitourinary (GU) adverse effects. No intraoperative or perioperative complications occurred.

**Conclusions:**

For selected patients with low- or intermediate-risk localized prostate cancer, F-BT is a safe and effective therapy.

## Introduction

According to the German S3 (AWMF) and many international guidelines (EUS, ESTRO, ABS, NCCN), brachytherapy is part of the radiotherapeutic treatment options for prostate cancer (PC) with low or intermediate risk. LDR-BT with permanently implanted radioactive seeds is recommended only for the low-risk PC group with localized states. As monotherapy, LDR-BT is a successful and well-tolerated treatment for men with low- and intermediate-risk prostate cancer (Leitlinienprogramm Onkologie 2022; Mottet et al. 2020; Yamada et al. 2012; NCCN 2023). HDR-BT can be offered in both locally limited and locally advanced intermediate- and high-risk PC in combination with percutaneous radiotherapy (Mottet et al. 2020).

HDR brachytherapy as monotherapy is recommended in the AWMF guidelines only within trials. In contrast, the joint guideline of the EUS, ESTRO, etc. (Mottet et al. 2020) and the recommendations of the NCCN (2023) allow this option for patients with low or intermediate risk. HDR may provide radiobiological benefits in malignancies with low alpha/beta ratios. Since HDR is scheduled after catheter insertion, it eliminates dosage uncertainty from seed movement or misplacement.

Several institutional series indicate high cancer control rates for individuals with low- and intermediate-risk illness following 4 or 6 portions of HDR monotherapy (Mottet et al. 2020). Authors were utilizing 3- or 2-fraction regimens with a larger dosage per fraction report comparable control rates but with a shorter median time of follow-up. The actual NCCN guidelines offer a treatment schedule of 13.5 Gy × 2 fractions as a standard HDR monotherapy regimen (NCCN 2023). 20 Gy as a single fraction would produce the exact physiologically equivalent dosage as 13.5 Gy × 2, based on radiobiological calculations utilizing an alpha/beta ratio of 3.0 for prostate cancer.

Several single-institution investigations utilizing a single fraction of 19–20.5 Gy have demonstrated short-term clinical results (Prada et al. 2016, 2019, 2020; Peters et al. 2019). If successful and safe, single fraction HDR might offer significant advantages over fractionated regimens regarding resource usage, cost, and patient comfort.

Given the potential benefits of single fraction HDR, we undertook a prospective feasibility study administered (only) as 20 Gy of HDR administered in a single fraction. The cited guidelines emphasize the potential advantages of focal therapy in local limited PC with low or intermediate risk in terms of reduction of side effects (ref. HIFU). Furthermore, irradiation with HDR-BT could significantly decrease the dose exposition of the adjacent organs at risk. However, there is no recommendation to use focal HDR-BT outside prospective studies due to a lack of significant study data.

Our hypothesis was that focal HDR-BT with a single dose of 20 Gy would be efficacious and well tolerated. Our primary aim was to assess the impact on health-related quality of life at 12 months. The secondary aims of this study were to evaluate acute and late toxicity to guide a subsequent more significant randomized phase III trial to verify biochemical and clinical disease control rates after these treatment procedures.

## Materials and methods

The Research Ethics Board in the medical faculty of the university Magdeburg registered the clinical trial. Eligible patients had histologically proven prostate adenocarcinoma, clinical stage T1c or T2a, a Gleason Score of 6 or 7, and a serum prostate-specific antigen (PSA) level of less than 20 ng/mL. Participants were required to provide informed permission and be able and willing to complete the Quality-of-Life Questionnaires. Quality-of-life assessments were performed using the international prostate symptom score (IPSS), the International Index of Erectile Function (IIEF-6), the International Consultation on Incontinence Questionnaire-Short Form (ICIQ-SF-2004), and the quality of life of cancer patients.

### Questionnaire (EORTC-QLQ-C30)

Exclusion criteria included evidence of distant or lymph-node metastases, prior pelvic radiotherapy, prior trans-urethral resection of the prostate, use of androgen deprivation therapy, an International Prostate Symptom Score (IPSS) of > 18, connective tissue or inflammatory bowel disease, and significant medical comorbidity rendering the patient unsuitable for general anesthesia. Block randomization was utilized to assign subjects to one of the two treatment groups. Physical examination, evaluation of baseline toxicity using Common Terminology Criteria for Adverse Events (CTCAE) v4.0, and completion of the IPSS (Strouthos et al. 2013) questionnaires comprised the baseline evaluation.

The treatment consisted of 20 Gy of HDR administered in a single fraction. To identify the Urethra, a 12-French urinary catheter was placed before the start of application. The patients were positioned in the prone position. After local anesthesia, median 2 [range 1–3 brachytherapy catheters (Premed Halberstadt, Germany) were placed transgluteal using 3T-MRI fluoroscopy guidance within the tumor lesions in freehand technique. For the detailed description of the catheter application, reference is made to the work of Fischbach et al. (2020).

The MRI was utilized to delineate the prostate, tumor [gross tumor volume (GTV)], and organ at risk (OAR). Along the length of the prostate, the prostate, the outside surface of the catheter, bladder, and the rectum were contoured. The clinical target volume, or CTV, were calculated by adding a 3 mm buffer around the GTV inside the prostate. There was no margin for CTV to PTV, since the source and dosage distribution moved with the tumor.

To assess feasibility, a pre-treatment plan was developed. The MRI procedure included transversal T2-weighted turbo spin echo (TSE) pictures with a resolution of 0.5 × 0.5 × 4 mm^3^ (for anatomical imaging). T2-weighted images were acquired with a slice thickness of 3 mm and an in-plane resolution of 0.5 mm 0.5 mm for the best signal-to-noise ratio and resolution balance. 2D T2 imaging was chosen for catheter reconstruction and delineation.

The prescription dose (20 Gy) was prescribed to the PTV in accordance with the following planning objectives: D100 > 95%; D90 > 100%, rectum D2cc < / = 4.5 Gy; bladder D2cc ≤ 6.2 G; Optimization of dwell duration was achieved using a dose–volume histogram (DVH). After treatment, BT catheters were withdrawn, the patients were turned into a supine position. Urinary catheter could be removed within the next hours if no signs of bleeding were evident. Patients were released home the next day.

Data were collected before the Intervention and at the 6-, 12-, and 24-month follow-ups. At each visit, serum PSA, toxicity, and IPSS were assessed. The patient completed-questionnaires at 6 months, 12 months, and yearly thereafter. Furthermore, mp-MRI was scheduled at 6, 12, and 24 months, as well as a re-biopsy 12 months after the focused HDR brachytherapy.

Failure (LF) was defined as the presence of biopsied PCa within the treated volume. A transperineal prostate biopsy was performed on participants whose PSA increased during follow-up. Active monitoring, salvage focused therapy, and radical therapies like as surgery or external beam radiation therapy were available following failure or relapse. A recurrence within or outside the field, salvage therapy was provided.

### Considerations based on statistics and sample size

Simple descriptive statistics are employed to summarize baseline clinical, demographic, and treatment planning conducted using Wilcoxon’s rank-sum or Fisher’s exact test. IPSS changes over time were analyzed using general linear regression. Within the first 3 months of therapy, toxicity was considered acute.

## Results

Between January 2018 and April 2021, 37 patients were assigned to receive 20 Gy in 1 fraction. The median duration of follow-up is 20 months (range 14–48) (Table 1). The median PSA was 8.24 ng/ml (range 2.4–23.70 ng/ml) and the median age was 66.5 years (range 52–80 years). 18/37 had Gleason 6 on average, whereas 17/37 had Gleason 3 + 4 and only 2 patients had Gleason 4 + 3. The median number of catheters utilized was two (range 1–3). Comparable relative dosimetry was seen, with a median V100 of 98% (97–99%) and a median D90 of 109%. An achieved median D100 for the PTV were 20.3 (range 11.4–21.9), for GTV D100 were 23.25 (range 15.6–32.4) (Table 2).

**Table 1:** Patient and tumor features

	Nr	Range	Percent	Median	Mean
Age		52–80		66.5	67.4
Initial PSA		2.4–16.8		8.24	8.24
Gleason grade					
6	18/37				
7a	17/37				
7b	2/37				
Clinical T stage					
T1c	13/37				
T2a	14/37				
Clinical D’Amico risk					
Low	19/37				
Intermediate	18/37				
Functional aspects					
IPSS		0–12		6.5	7.13
IIEF-5		6–30		22	20.94
ICIS		0–12		0	1.38
Treatment aspects					
GTV (cc)		0.3–6		2.04	2.58
PTV					
Prostata (cc)		21.5–95.7		39.76	44.5
Needles		(1–3)		2	2
BT-duration (s)		246–1292		570	605

**Table 2:** Dosimetric data

Items	Median	[Min–max]
GTV (cc)	2.04	[0.3–6]
D100% (%)	115	[78–160]
EQD2D100 (Gy)	122	[58–236]
V100% (%)	99	[96–99]
PTV (cc)		
D100% (%)	100	[57–109]
EQD2D100 (Gy)	122	[32.94–109]
Bladder		
D2cc (Gy)	8.4	[2.03–21.13]
EQD2D2cc (Gy)	19.15	[2.04–101.97]
Rectum		
D2cc (Gy)	5.7	[1.93–12.37]
EQD2D2r (Gy)	9.92	[1.9–38.03]

### Dosimetry

The median PTV was 5.015 ml, mean 6.16 ml (range 1.67–12.25 ml), while median GTV was 2.04 ml (range 0.30–2.58). The median volume of the Prostate was 36.5 ml (range 18–101 ml) (Fig. 5). A median of 2 HDR catheters were placed (range 1–3). The median time of brachytherapy was 570 s (range 246–1292 s). For the OAR, median dose to 2 cc Rectum was 5.68 Gy (range 1.93–12.3 Gy), while median D2cc Blasé was 8.4 Gy (range 2.03–21.13 Gy). The median Dmax for the extraprostatic Urethra was 8.49 Gy (range 2.97–25.32 Gy). After 3 months, median PSA was 2.8 ng/ml, mean 3.2 (range 0.7–10.8 ng/dl), and after 6 months, median PSA was 2.3 ng/ml (range 0.4–10.8), while after 24 months, median PSA was 1.9 ng/dl (range 0.5–8.7 ng/dl).

### Response

The 2-year overall survival rate was 100%, 2-year BCR-free survival rate was 100%, and 2-year LF-free survival rate was 89% (Fig. 3). After 24 months, a systematic control biopsy was done on 35 patients, and only six patients had a positive “in-field” biopsy. Clinical T stage, Gleason score, and pre-treatment PSA, age, and brachytherapy dosage were the factors that were employed in multiple regression analyses to determine the characteristics that correlated with biochemical failure. There were no independent predictive variables for biochemical failure found in the multivariate Cox regression models (Table 3, Fig. 4).

**Table 3:** Functional aspects differences during the follow-up

Overall cohort	Mean	*P* value
*N*: 37		
IPSS		
Baseline vs 6 months	7.1 vs 7.7	0.11
Baseline vs 12 months	7.1 vs 7	0.23
Baseline vs 24 months	7.1 vs 7.0	0.19
IIEF-5		
Baseline vs 6 months	20.9 vs 20.1	0.13
Baseline vs 12 months	20.9 vs 19.9	0.45
Baseline vs 24 months	20.9 vs 20.0	0.27
ICIQ		
Baseline vs 6 months	1.4 vs 1.5	0.31
Baseline vs 12 months	1.4 vs 1.8	0.17
Baseline vs 24 months	1.4 vs 1.7	0.23
Baseline vs 24 months	7.1 vs 7.0	0.19

A PSA bounce occurred in six patients. The term “PSA bounce” refers to a spike in serum PSA of at least 0.2 ng/ml over the nadir level followed by a reduction in PSA. PSA bounce peak was 0.6 ng/mL on average (range 0.2–1.2 ng/ml). The median time to bounce was 6 months (range 3–12 months).

### Toxicity

#### Acute toxicity

There are follow-up data on 37 patients at 3 months. There were no notable acute gastro-intestinal (GI) adverse effects. One instance of diarrhea, proctitis, and hemorrhoids of grade 2 were the most severe cases of acute toxicity documented. Genito-urinary (GU) toxicity was more prevalent. In the acute phase, five patients experienced acute urinary retention grade 2 after treatment; however, none of these patients required intervention, all were treated with the use of alpha-blockers. One patient had acute grade 2 hematuria soon after the treatment, necessitating hospitalization overnight for bladder irrigation.

#### Chronic toxicity

There are follow-up data for at 6, 9, 12, and 24 months, respectively. No further new grade 2 or higher GI or GU toxicity was identified during a median follow-up of 24 months (range 1–36 months). 32/37 patients had no change in erectile function. When compared to baseline, 5 patients had a slight deterioration in erectile function.

### Urinary complaints

**Fig. 1:**
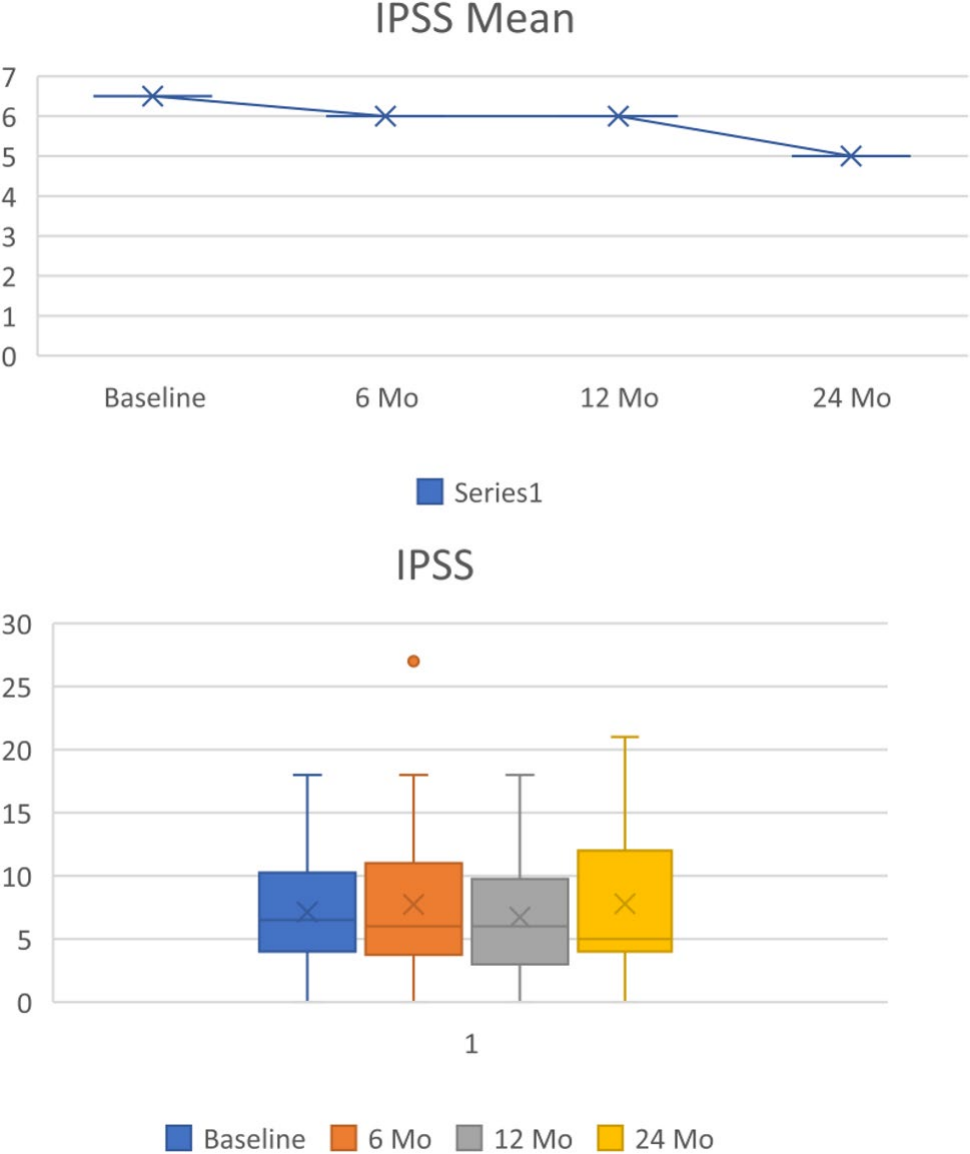
IPSS

At baseline, 6 months, 12 months, and 24 months, the median IPSS was 6.5, 6.6, and 5, respectively. At 6 months, the IPSS remained statistically considerably similar as at baseline, although changes did not achieve statistical significance at later time points. In the first 12 months, the proportion of patients reporting no, or light urine symptoms (IPSS 6–7) was considerably lower than at baseline (Figs. 1 and 3).

### Sexual function

**Fig. 2:**
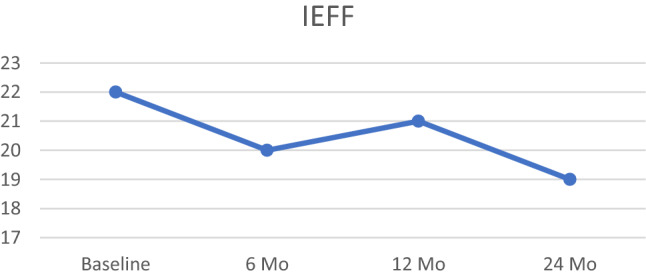
IEFF

Erectile function was evaluated at the baseline using IIEF-6, score was 20.9 (range 6–30). Over the first 24 months in our group, the mean IIEF-6 scores slightly decreased. The mean IIEF-6 score at 6, 12, and 24 months to 20.1, 19.8, and 19.7, respectively (Fig. 2).

## Discussion

Although most of the published data supporting the use of HDR as monotherapy have used four or more fractions, there is the radiobiological rationale for investigating more hypofractionated regimens (Prada et al. 2016, 2019, 2020; Demanes and Ghilezan 2014; Hoskin et al. 2017a; Morton et al. 2017; Strouthos et al. 2018; Yoshioka et al. 2003). To determine optimal HDR dose and fractionation, Mavroidis et al. conducted detailed radiobiological modeling of tumor control and normal tissue complication probability based on three-dimensional dose–volume histograms of HDR implants using different fractionation schemes (Mavroidis et al. 2014). Using four fractions of 9.5 Gy as standard, their model predicted that either two fractions of 13.2–13.8 Gy or a single fraction dose of 19.2–19.7 Gy would have as good or better tumor control and lower normal tissue complications. They predicted that a single fraction of 19.5 Gy would result in a 98.5% tumor control and a 40–55% reduction in the probability of complications compared to the standard four-fraction regimen. These models, however, have several limitations including an inability to account for a potential effect of hypoxia and reoxygenation.

**Fig. 3:**
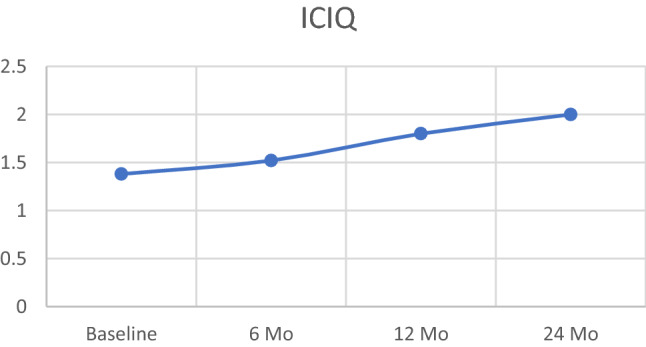
ICIQ

**Fig. 4:**
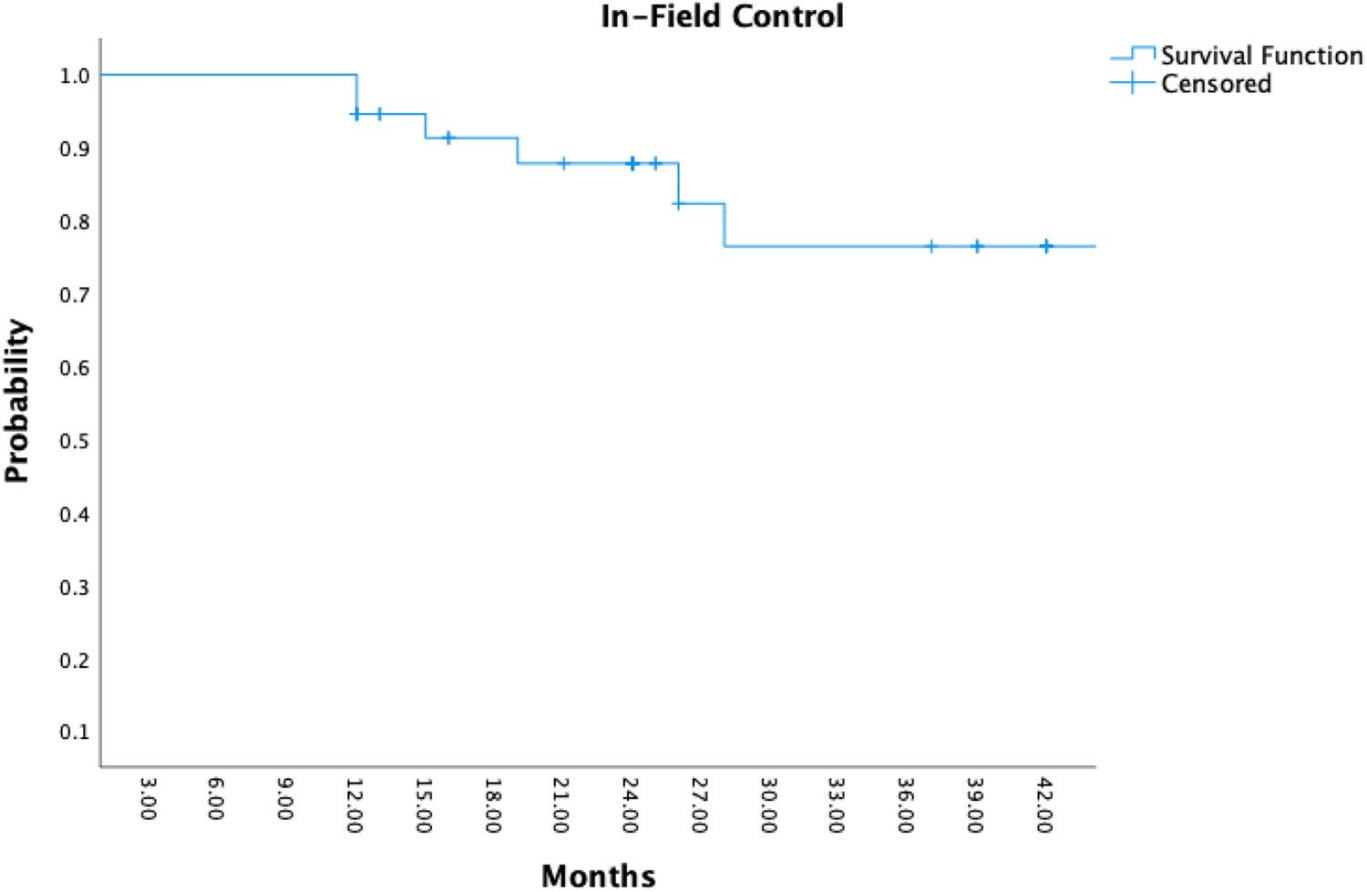
LF in Kaplan–Meier

**Fig. 5:**
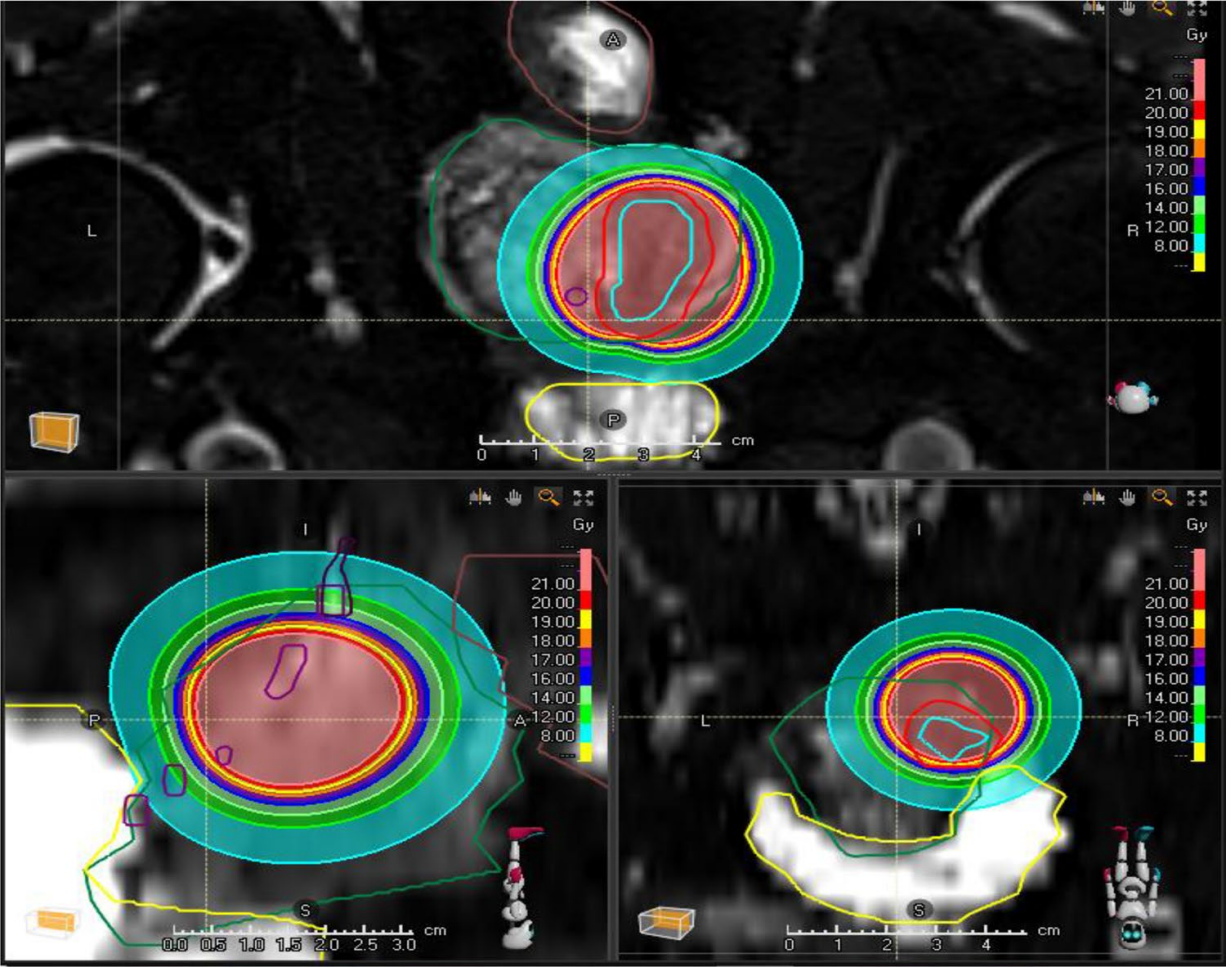
Focal brachytherapy with 1 × 20 Gy

Recent clinical data are available on well-fractionated HDR regimens. Delivering a median dose of 43.5 Gy in six fractions and a median follow-up of 6.5 years, investigators at UCLA report a 10-year biochemical disease-free survival of 97.8% for 448 patients with low- and intermediate-risk disease (Hauswald et al. 2016). The authors found no grade 3 rectal toxicity and a 4.7% rate of grade 3 urinary toxicity. The largest HDR monotherapy series in the literature comes from Offenbach, which included 492 patients treated to 38 Gy in four fractions and 226 treated to 34.5 Gy in three fractions (Zamboglou et al. 2013). Biochemical disease-free survival at 5 years was 98% and 95%, respectively. Although there was no reported clinical outcome difference between the two fractionation schemes, median follow-up was only 25 months for the three-fraction regimen and 59–92 months for the four-fraction regimen.

To date, there are limited clinical data on more hypofractionated regimens, with somewhat conflicting results. Prada et al. were the first to publish outcome data following single fraction 19 Gy as monotherapy in a series of 40 patients with low- and intermediate-risk disease (NCCN 2023). No grade 2 or higher toxicity was observed, and only one patient (2.5%) developed acute urinary retention requiring a catheter. An update of the series has been reported, now including 60 patients—44 low-risk and 16 intermediate-risk (Hoskin et al. 2017a). With a median follow-up of 72 months, the 6-year biochemical disease-free survival was only 66%. While it may be assumed that the high biochemical failure rate related to colder implants, the investigators discovered no connection between D90 and recurrence risk.

Hoskin et al. have studied increasingly more hypofractionated HDR monotherapy regimens in patients with locally advanced prostate cancer, nearly usually accompanied with some length of androgen restriction treatment. An initial regimen of 10.5 Gy × 3 (*n* = 109) was compared with 13 Gy × 2 (*n* = 118) (Hoskin et al. 2017b). The two-fraction regimen was related with decreased grade 1 and 2 bowel and urine toxicity. With a median follow-up of 71 months for the three-fraction treatment and 31 months for the two- fraction regimen, the 3-year biochemical failure-free survival was 97% and 93%, respectively. Hoskin also observed acute toxicity results comparing patients treated to 13 Gy × 2 with a further 20 patients treated to 19 Gy × 1 and 26 patients treated to 20 Gy × 1. Patients treated to 20 Gy had more severe urinary.

Our report is one of the first prospective German trials to look at the feasibility of HDR focal brachytherapy as a single treatment for patients with favorable-risk prostate cancer. The data suggest that F-BT is possible, safe, and effective for highly selected patients with low- to intermediate-risk localized PCa and acceptable late urinary and sexual toxicity rates. Compared to other focal therapies, F-BT has better conformality and makes it possible to increase the radiation dose in the treated volume (Chargari et al. 2019). F-BT also gives 2 to 3 mm of coverage around the periprostatic margin outside of the capsule, which is good enough from an oncological point of view (Crook et al. 2010). F-BT is interesting for peripheral lesions, because it has these technical advantages over other types of focal therapy; the radioactive coverage can be “tailor-made”, because the catheter placement can be changed to fit the size and location of the tumor (Sivaraman and Barret 2016). This study showed that the midterm clinical results of F-BT were the same as those of whole-prostate brachytherapy.

The current findings are encouraging in terms of both chronic toxicity and quality of life. However, more study of these patients is required before more conclusive findings can be reached. Toxicity rates are greater with whole gland treatments, such as low-dose-rate brachytherapy (Stone and Stock 2002). The prevalence of acute urinary retention, the most prevalent grade 2 toxicity, ranges from 5 to 34% (Stone and Stock 2002; Roeloffzen et al. 2011). However, acute urine retention (Hauswald et al. 2016) is not recorded in a recent series with a 10-year follow-up. Nonetheless, the present study only showed short-term findings in a small patient population after a median of 24 months of follow-up, which may underestimate toxicity. If toxicity remains as minimal as observed, normal tissue dosage prescription may be less stringent. The literature on focused treatment is currently limited (Ahmed et al. 2015; Nguyen et al. 2012; Laing et al. 2016).

During follow-up, there was no statistically significant difference in quality of life compared to baseline in any of the surveys. This might be explained by the comparatively low number of patients at risk at this time, as well as the low toxicity rate. There were changes of more than 10 points noted throughout the duration of the follow-up, e.g., social functioning and pain 1 month after therapy, compared to baseline. This has been reported as a clinically meaningful rise or reduction in one series (Osoba et al. 1998).

According to our knowledge, this is one of the limited studies that present the midterm results of F-BT in PCa patients with low-to-moderate risk who were carefully chosen. However, there are a few limitations, such as the limited number of patients, the absence of randomization, and the short follow-up period, despite the fact that our midterm findings have been sufficiently validated. In addition, this research was developed at the beginning of focused treatment, which explains why the majority of patients recruited had prostate cancer with an excellent prognosis. In the future, these midterm results should be verified by other prospective multicenter trials including a greater number of patients with PCa at intermediate risk.

## Conclusion

In comparison to alternative focal therapies, the research indicates that F-BT is feasible, safe, and effective for carefully chosen individuals with low- to intermediate-risk localized PCa and with tolerable rates of genital and urinary toxicity. It is a good approach for PCa focal treatment, because, among other benefits, it allows for the potential of adjusting the implantation to the volume and location of the tumor.

## Data Availability

The datasets used and analyzed during the current study are available from the first author on reasonable request.

## Abbreviations

PCa: Prostate cancer
F-BT: Focal brachytherapy
IPSS: International Prostatic Symptom Score
PSA: Prostate-specific antigen
LDR-BT: Low-dose-rate brachytherapy
mp-MRI: Multiparametric magnetic resonance imaging
IIEF-5: International Index of Erectile Function
